# Association Between Secondary Hyperparathyroidism and Body Composition in Pediatric Patients With Moderate and Advanced Chronic Kidney Disease

**DOI:** 10.3389/fped.2021.702778

**Published:** 2021-08-12

**Authors:** Vasiliki Karava, Antonia Kondou, John Dotis, Athanasios Christoforidis, Anna Taparkou, Konstantina Tsioni, Evangelia Farmaki, Konstantinos Kollios, Ekaterini Siomou, Vassilios Liakopoulos, Nikoleta Printza

**Affiliations:** ^1^Pediatric Nephrology Unit, First Department of Pediatrics, Hippokratio General Hospital, Aristotle University of Thessaloniki, Thessaloniki, Greece; ^2^Pediatric Endocrinology Unit, First Department of Pediatrics, Hippokratio General Hospital, Aristotle University of Thessaloniki, Thessaloniki, Greece; ^3^First Department of Paediatrics, Pediatric Immunology and Rheumatology Referral Center, Hippokratio General Hospital, Aristotle University of Thessaloniki, Thessaloniki, Greece; ^4^Biopathology Laboratory, Hippokratio General Hospital, Thessaloniki, Greece; ^5^Third Department of Pediatrics, Hippokratio General Hospital, Aristotle University of Thessaloniki, Thessaloniki, Greece; ^6^Department of Pediatrics, University Hospital of Ioannina, Ioannina, Greece; ^7^Division of Nephrology and Hypertension, First Department of Internal Medicine, AHEPA Hospital, Aristotle University of Thessaloniki, Thessaloniki, Greece

**Keywords:** secondary hyperparathyroidism, parathormone, muscle wasting, fat, leptin, chronic kidney disease, alfacalcidol, children

## Abstract

**Objective:** This single center cross-sectional study aims to investigate the association between secondary hyperparathyroidism and body composition in pediatric patients with moderate (stage 3) and advanced (stage 4–5) chronic kidney disease (CKD).

**Methods:** 61 patients (median age: 13.4 years) were included. Body composition indices, including lean tissue index (LTI) and fat tissue index (FTI), were measured using multi-frequency bio-impedance spectroscopy. Muscle wasting was defined as LTI adjusted to height-age (HA) z-score < −1.65 SD and high adiposity as FTI z-score > 1.65 SD. Serum mineral metabolism parameters, including serum intact parathormone (iPTH), calcium, phosphorus and 25-hydroxyvitamin D, as well as serum leptin were measured in each patient. In advanced CKD patients, the mean values of serum mineral laboratory parameters of the 6 months prior to body composition assessment were recorded, and alfacalcidol index, defined as weekly alfacalcidol dose (mcg/week) per pg/ml of iPTH × 1,000, was calculated.

**Results:** In moderate CKD (31 patients), high iPTH (>90 ng/ml) was observed in 10 (32.3%) patients and was associated with higher FTI z-score (*p* = 0.022). Moreover, serum iPTH was negatively correlated to LTI HA z-score (rs = −0.486, *p* = 0.006), and positively correlated to serum leptin levels (rs = 0.369, *p* = 0.041). The positive correlation between FTI z-score and iPTH (rs = 0.393, *p* = 0.039) lost significance after adjustment for serum leptin. iPTH was positively associated with high adiposity (12 patients, 38.7%) after adjustment for the other mineral metabolism parameters (OR 1.023, 95% CI 1.002–1.045, *p* = 0.028). In advanced CKD (30 patients), no significant correlation was observed between iPTH and body composition indices and serum leptin levels. Eleven (36.7%) patients with muscle wasting presented lower alfacalcidol index (*p* = 0.017). Alfacalcidol index ≤ 24 was strongly associated with muscle wasting after adjustment for CKD stage and other mineral metabolism parameters (OR 7.226, 95% CI 1.150–45.384, *p* = 0.035).

**Conclusion:** Secondary hyperparathyroidism is associated with high adiposity in moderate but not in advanced CKD, with leptin acting as a potential contributive factor. In advanced CKD, targeting higher alfacalcidol weekly dose per each unit of serum PTH seems beneficial for preventing muscle wasting.

## Introduction

Chronic kidney disease (CKD) is generally considered as a chronic catabolic disease, resulting in increased muscle protein and fat metabolic rate. Muscle wasting, involving loss of muscle mass and/or muscle strength, is present even in the early course of the disease, while its prevalence rises with decline of kidney function, ultimately affecting more than 40% of pediatric patients with advanced CKD (1, 2). Multiple mechanisms have been implicated in its pathogenesis, including anemia, chronic metabolic acidosis, anorexia, increased oxidative stress, accumulation of inflammatory cytokines and endocrine disorders, such as disturbed growth hormone/insulin growth factor-1 signaling (3). Fat loss is less frequently marked in pediatric patients and is predominantly encountered in advanced CKD. Additionally, increased adiposity, primarily attributed to reduced physical activity and adoption of high-fat western diet, is increasingly observed in pediatric CKD patients, with a prevalence of overweight and obesity among European pediatric patients on renal replacement therapy of 20.8 and 12.5%, respectively (4). Both muscle wasting and high adiposity, which may be coupled especially in case of normal-weight obesity, have been proven unfavorable prognostic factors for the patient overall morbidity. Muscle wasting eventually leads to protein energy wasting and frailty phenotype, which may compromise patient life quality, deteriorate bone disease (5, 6) and increase hospitalization (7) and mortality risk (8), while high body adiposity levels have been associated with aggravated metabolic and cardiovascular risk (9–11), more rapid CKD progression (12) and reduced access to kidney transplantation (13). Therefore, early recognition of body composition disturbances and prompt management of the related pathogenetic risk factors as well as the subsequent adverse patient outcomes are crucial for the optimal healthcare delivery of these patients.

Nowadays, increasing evidence suggest that fat and muscle tissue are considered as endocrine organs, while adipokines and myokines facilitate their pleiotropic endocrine activity. Among the interactions between fat and muscle tissue and various metabolic pathways, results from experimental and clinical studies indicate a direct link between serum parathyroid hormone (PTH) and fat and muscle metabolism and a possible contributive role of adipocyte derived leptin on the crosstalk between PTH and fat status. It is current knowledge that obesity is commonly linked to higher serum PTH levels, in both general adult (14) and pediatric population (15), ascertaining a direct endocrine crosstalk between adipose tissue and parathyroid gland. This finding was also confirmed in CKD patients, where secondary hyperparathyroidism (SHPT) was more frequently observed in obese pre-dialysis adult patients (16, 17). Similar data in pediatric population are limited to kidney transplant patients, in whom high body mass index (BMI) was positively associated with SHPT in a recent cohort study (18). Besides, lower serum PTH levels in advanced CKD adult patients may reflect poor nutritional status, which in turn, increases mortality risk (19, 20). Nevertheless, *in vivo* studies in CKD mice have recently pointed out that SHPT may promote adipose tissue browning and muscle wasting, through stimulation of thermogenic gene expression, and more precisely of uncoupling protein-1 (Ucp1), ultimately leading to cachexia (21, 22). Data from clinical studies investigating the possible lipolytic and muscle proteolytic effects of high PTH are lacking and primarily concern adult population, where severe SHPT negatively affects body adiposity levels in CKD patients requiring parathyroidectomy (23, 24). The aim of this study was to investigate the association between body composition, involving both body fat and muscle status, using multi-frequency bioimpedance spectroscopy (BIS) technique, and serum leptin with serum PTH status in both moderate and advanced CKD pediatric patients.

## Materials and Methods

We conducted a cross-sectional study on the data of children and adolescents with CKD followed-up at the 1st and 3rd Department of Pediatrics at the Hippokratio General Hospital of Thessaloniki from March 2018 to December 2020. Inclusion criteria included: (i) age of participants between 5 and 19 years old and (ii) estimated glomerular filtration rate (eGFR) <60 ml/min/1.73 m^2^, as calculated by the revised Schwartz formula. Moderate CKD was defined as the presence of CKD stage 3 and advanced CKD as the presence of CKD stage 4 to 5, including patients on chronic dialysis (CKD 5D).

Anthropometric data, including weight, height and body mass index (BMI), were converted into z-scores based on Centers for Disease and Control and Prevention (CDC) reference values for healthy children of the same age and sex (25). BMI z-score adjusted to height age (BMI HA z-score) was calculated for all patients. Patient body composition was estimated by multi-frequency BIS technique, using the same portable BIS device (Body composition monitor, BCM, Fresenius Medical Care, Bad Homburg, Germany). Both anthropometric and body composition measurements were effectuated on the same day by the same physician. Participants with CKD 3-4 were instructed to refrain from eating and drinking for at least 8 h prior to testing, while body composition assessment was obtained at the estimated dry weight in CKD 5D patients. In detail, BIS measurement was carried out 3 h post dialysis session and after drainage of peritoneal fluid in patients on peritoneal dialysis, and 1 h after the end of a mid-week hemodialysis session in patients on hemodialysis. Muscle mass and fat mass status were estimated using lean tissue index (LTI) and fat tissue index (FTI), defined as lean mass to height^2^ (kg/m^2^) and fat mass to height^2^ (kg/m^2^), respectively, which were derived from the BIS device impedance data. LTI to FTI ratio (LTI/FTI) was also calculated for each patient. All LTI and FTI values were converted into z-scores using body composition reference curves for healthy children in the United Kingdom (UK) (26). Muscle wasting and reduced adiposity were defined as the presence of LTI adjusted to height age z-score (LTI HA z-score) and FTI z-score lower than the 5th percentile (z-score < −1.65 SD), while high LTI and increased adiposity as the presence of LTI adjusted to height age z-score (HA) (LTI HA) and FTI z-score higher than the 95th percentile (z-score > 1.65 SD).

Serum mineral metabolism parameters, including serum intact parathormone (iPTH), calcium (Ca), phosphorus (P) and 25-hydroxyvitamin D [25(OH)D], which were analyzed at the same local hospital laboratory using standard commercial assays, were collected for all patients on the day of body composition assessment. In moderate CKD, high iPTH was defined as iPTH > 90 ng/ml, based on local laboratory reference ranges and the highest tercile of iPTH values in this patient group. In patients with advanced CKD, mean values of serum mineral metabolism parameters were recorded for a 6-month period prior to body composition assessment. All patients with advanced CKD had a minimum of 3 measurements of serum mineral metabolism parameters during the study period. Alfacalcidol treatment was administered in all patients with advanced CKD, in form of drops or capsules, while none of the patient with moderate CKD was treated. The 6-month mean daily alfacalcidol dose was calculated. Moreover, alfacalcidol index, defined as 6-month mean alfacalcidol dose (mcg/week) per pg/ml of iPTH × 1,000, was calculated in advanced CKD patients. Of note, none of the patients was under calcimimetic treatment during the study period. Finally, serum leptin levels, using ELISA technique (R&D systems, Minneapolis, MN, USA) was measured in all patients on the day of body composition assessment.

Data were expressed as median values and ranges. All statistical tests were performed using IBM corp. SPSS Statistics® software for Windows. Mann Whitney and Fisher's exact and multiple chi-square tests were used to compare the distribution of the on-study continuous and categorical parameters between different patient groups. Spearman correlation tests were used to assess the univariate correlations between the on-study study parameters, whereas spearman partial correlation analysis was used to assess the correlations between the on-study variables after adjustment for possible confounders. Univariate and backward multiple logistic regression were performed in order to detect association between mineral metabolism profile and increased adiposity in moderate CKD and muscle wasting in advanced CKD. A *p*-value of <0.05 was considered statistically significant.

## Results

Sixty-one patients, 40 males and 21 females, with a median age of 13.4 years (range 5.2–19.7), were included in this study. Etiology of CKD included congenital abnormalities of kidney and urinary tract in 39 (63.9%), ciliopathy in 6 (9.8%), hemolytic uremic syndrome in 5 (8.2%), focal segmental glomerulosclerosis in 3 (5%), congenital nephrotic syndrome in 2 (3.3%), chronic interstitial nephritis in 1 (1.6%), familial hyperuricemic nephropathy in 1 (1.6%) and of unknown origin in 4 (6.6%) patients, respectively. CKD stage 3 was present in 31 (50.8%) patients, stage 4 in 13 (21.3%) patients, while 17 (27.9%) patients were on chronic dialysis; 14 on peritoneal dialysis and 3 on hemodialysis. Of note, 8 patients were previously kidney transplanted with a median duration from kidney transplantation of 7.1 (5.7–8.9) years.

**Figure 1:**
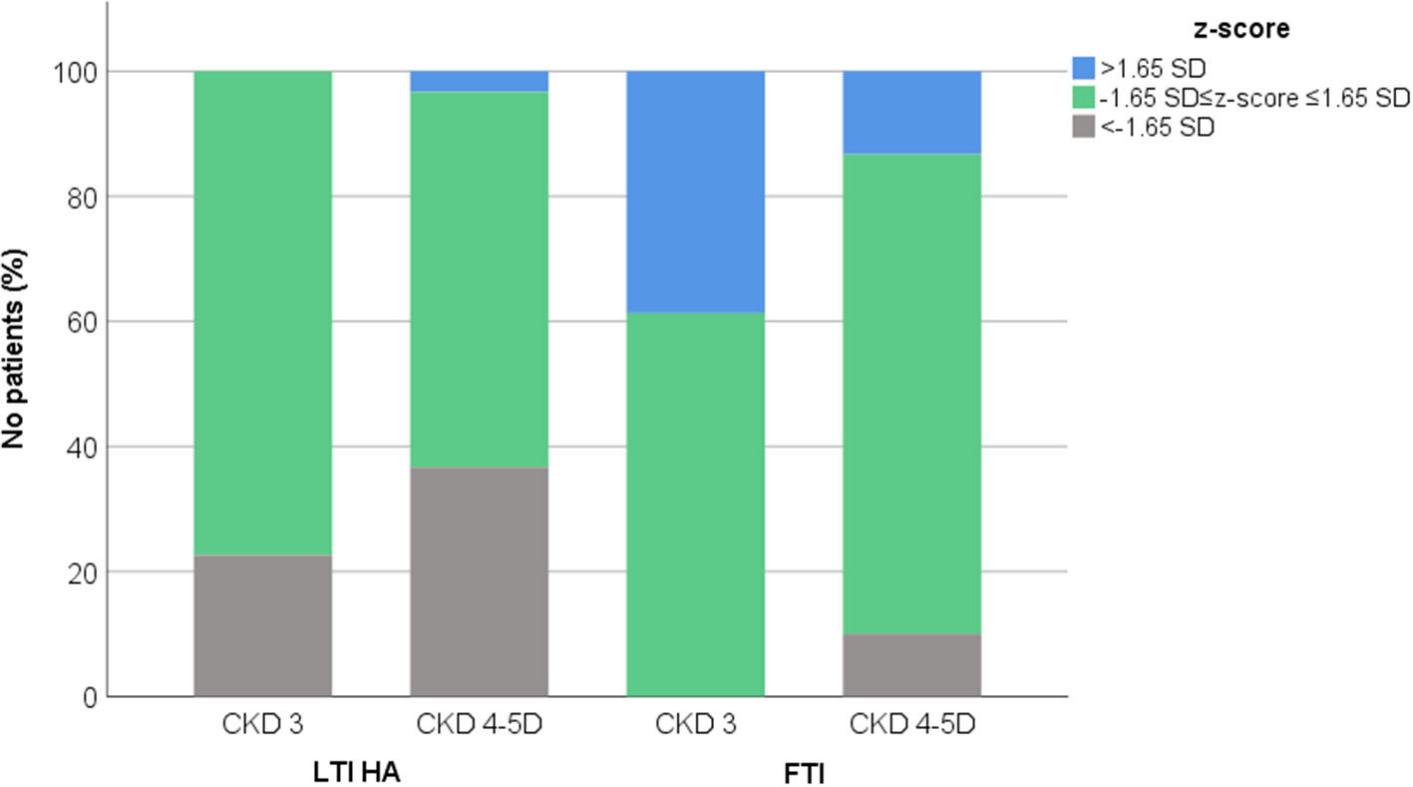
Distribution of lean tissue index adjusted to height-age (LTI HA) and fat tissue index (FTI) z-scores in chronic kidney disease (CKD) stage 3 and 4–5D patients.

Patients were divided according to CKD stage, in those presenting moderate CKD (stage 3, 31 patients) and advanced CKD (stages 4 and 5D, 30 patients). Distribution of anthropometric parameters, body composition indices and laboratory data in patients with moderate and advanced CKD are illustrated in Table 1. In total, LTI HA z-score was within normal range in 24 (77.4%) patients with moderate and in 18 (60.0%) patients with advanced CKD, while low LTI HA z-score (<-1.65 SD) was observed in 7 (22.6%) patients with moderate and in 11 (36.7%) patients with advanced CKD (Figure 1). High FTI z-score (>1.65 SD) was observed in 12 (38.7%) patients with moderate and in 4 (13.3%) patients with advanced CKD. Of note, low FTI z-score (<-1.65 SD) was observed in only 3 (10%) patients with advanced CKD (Figure 1).

**Table 1:** Distribution of anthropometric parameters, body composition indices and laboratory data in chronic kidney disease (CKD) stage 3 patients with and without high iPTH (>90 ng/ml) and in CKD stage 4-5D patients.

	**CKD 3**				**CKD 4–5D**
	**Total** **31 patients**	**High iPTH** **10 patients**	**Normal iPTH** **21 patients**	**P**	**Total** **30 patients**
Age (years)	14.4 (6.5–18.8)	16 (10.7–18.8)	13.4 (6.5–18.7)	0.105	12.8 (5.1–18.1)
Sex (male)	19 (61.3%)	5 (50%)	14 (66.7%)	0.447	21 (70%)
Weight (kg)	54 (22.3–90)	56.6 (42–90)	50.6 (22.3–69)	0.053	34.2 (14–70)
Weight z-score	0.3 (−2.5–2.0)	1.1 (−1.8–2)	0.3 (−2.5–1.9)	0.233	−1.5 (−4.1–1.4)
Height (cm)	158 (123–178)	157 (133–175)	159 (123–178)	0.884	139.3 (93–177)
Height z-score	−0.4 (−2.9–2.1)	−1.1 (−2.9–1.6)	0.2 (−1.7–2.1)	0.025^**^	−1.43 (−5.71–1.1)
BMI (kg/m^2^)	21.1 (14.7–37.6)	25.6 (15.4–37.6)	20.1 (14.7–24.2)	0.003^**^	17.5 (14.3–22.7)
BMI z-score	0.6 (−2.8–2.5)	1.4 (−2.8–2.5)	0.3 (−2.1–2.1)	0.059	−0.5 (−3.1–1.5)
BMI HA z-score	0.8 (−2.1–2.5)	1.7 (−2.1–2.5)	0.3 (−1.5–2)	0.019^**^	−0.1 (−1.5–2.5)
LTI (kg)	13 (11–18.5)	12.6 (11–15.1)	13.3 (11.5–18.5)	0.250	13 (8.6–16)
LTI z-score	−1.3 (−3.4–0.5)	−2.3 (−3.4–0.4)	−1.3 (−3.2–0.5)	0.035^**^	−1.5 (−5.6–1.9)
LTI HA z-score	−1.1 (−3.1–1.1)	−1.3 (−3.1–0)	−0.9 (−2.4–1.1)	0.087	−1.3 (−4.9–2.4)
FTI (kg)	8.2 (1.7–26.7)	13.5 (2.3–26.7)	5.6 (1.7–10.9)	0.004^**^	4.4 (2.3–13.5)
FTI z-score	1.3 (−1.5–3.7)	2.1 (−0.6–3.5)	0.9 (−1.5–3.7)	0.022^**^	0.5 (−2.7–2.3)
LTI/FTI	1.6 (0.4–10.9)	0.9 (0.4–5.6)	2.6 (1.1–10.9)	0.007^**^	3.2 (0.6–6.6)
iPTH^*^ (pg/ml)	52.1 (11.1–176)	128.5 (91–176)	44.1 (11.1–87.5)	<0.001^**^	156.2 (53–650)
serum Ca^*^ (mg/dl)	9.7 (8.8–10.4)	9.6 (8.8–10.4)	9.8 (8.8–10.3)	0.348	9.6 (8.2–10.2)
serum P^*^ (mg/dl)	4.3 (3.4–5.5)	4.7 (4–5.5)	4.2 (3.4–5.2)	0.079	5.3 (3.4–7.3)
Serum 25(OH)D^*^ (ng/ml)	24.1 (12.5–37.6)	23.3 (12.5–28.6)	25.5 (18.9–37.6)	0.159	24.3 (12.3–54.9)
Serum albumin^*^ (g/dl)	4.4 (3.8–5)	4.3 (3.8–4.8)	4.4 (3.8–5)	0.917	4 (2.8–5)
Serum leptin (pg/ml)	10,890 (200–96,480)	45,270 (410–96,480)	6,250 (200–72,070)	0.022^**^	4,590 (270–116,700)

*BMI, body mass index; LTI, lean tissue index; HA, height-age; FTI, fat tissue index; iPTH, intact parathormone; Ca, calcium; P, phosphorus; 25(OH)D, 25-hydroxyvitamin D*.

* *In patients with CKD stage 4,5D the 6-month mean serum Ca, P, 25(OH)D and albumin were recorded*.

** *p statistically significant*.

**Figure 2:**
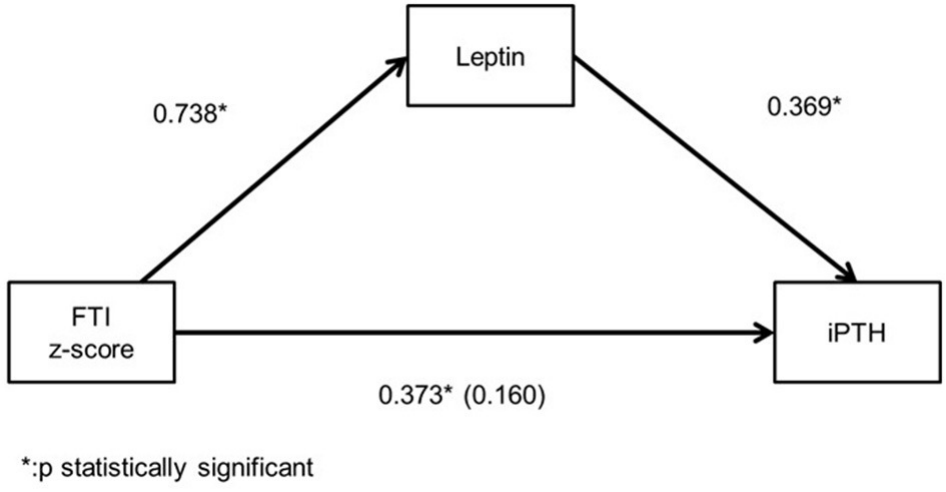
Mediation analysis illustrating the spearman's correlation coefficient between serum leptin, intact parathormone (iPTH) levels and fat tissue index (FTI) z-score and the partial spearman's correlation efficient (in parenthesis) between iPTH and FTI z-score after adjustment for serum leptin in chronic kidney disease (CKD) stage 3 patients.

Among the patients with moderate CKD, patients with serum iPTH >90 pg/ml (10, 32.3%) presented lower height and LTI z-scores (*p* = 0.025, *p* = 0.035, respectively), higher BMI HA and FTI z-scores (*p* = 0.019, *p* = 0.022, respectively) and lower LTI/FTI (*p* = 0.007) (Table 1). Accordingly, in spearman correlation analysis, serum iPTH was negatively correlated to height and LTI-z-scores (rs = −0.397, *p* = 0.027 and rs = −0.416, *p* = 0.020, respectively), positively correlated to FTI z-score (rs = 0.393, *p* = 0.039), and negatively correlated to LTI/FTI (rs = −0.450, *p* = 0.011) (Table 2). As expected, serum iPTH was positively correlated to serum P levels (rs = 0.465, *p* = 0.008), which were higher in patients with serum iPTH > 90 pg/ml, although the association did not reach statistical significance (*p* = 0.079) (Tables 1, 2). Moreover, serum leptin was positively associated with serum iPTH > 90 pg/ml (*p* = 0.022) and positively correlated to serum iPTH levels (rs = 0.369, *p* = 0.041). In mediation analysis, serum leptin was also positively correlated to FTI z-score (rs = 0.738, *p* < 0.001), while the correlation between FTI z-score and serum iPTH lost significance after adjustment for serum leptin (rs = 0.160, *p* = 0.398) (Figure 2). In univariate logistic regression analysis, serum iPTH was positively associated with high FTI z-score (>1.65 SD) (OR 1.024, 95% CI 1.004–1.044, *p* = 0.018) (Table 3) and in backward multiple logistic regression analysis the association remained significant after adjustment for other mineral metabolism laboratory parameters, including serum Ca, P and 25(OH)D (OR 1.023, 95% CI 1.002–1.045, *p* = 0.028).

**Table 2:** Spearman correlation analysis of anthropometric parameters, body composition indices and laboratory data with serum intact parathormone (iPTH) in chronic kidney disease (CKD) stage 3 patients and with 6-month mean iPTH and alfacalcidol index [(mcg/week per pg/ml of PTH) × 1,000] in CKD stage 4,5D patients.

	**CKD 3**	**CKD 4–5D**	
	**iPTH**	**iPTH**	**Alfacalcidol index**
Weight	rs = 0.141, *p* = 0.449	rs = 0.050, *p* = 0.793	rs = 0.407, *p* = 0.026^*^
Weight z-score	rs = 0.136, *p* = 0.465	rs = −0.233, *p* = 0.215	rs = 0.550, *p* = 0.002^*^
Height	rs = −0.090, *p* = 0.628	rs = 0.106, *p* = 0.576	rs = 0.379, *p* = 0.039^*^
Height z-score	rs = −0.397, *p* = 0.027^**^	rs = −0.188, *p* = 0.319	rs = 0.642, *p* < 0.001^*^
BMI	rs = 0.358, *p* = 0.048^**^	rs = −0.152, *p* = 0.422	rs = 0.302, *p* = 0.105
BMI z-score	rs = 0.253, *p* = 0.170	rs = −0.270, *p* = 0.149	rs = 0.166, *p* = 0.382
BMI HA z-score	rs = 0.310, *p* = 0.090	rs = −0.219, *p* = 0.246	rs = −0.025, *p* = 0.894
LTI	rs = −0.383, *p* = 0.034^**^	rs = 0.036, *p* = 0.849	rs = 0.591, *p* = 0.001^**^
LTI z-score	rs = −0.416, *p* = 0.020^**^	rs = −0.026, *p* = 0.893	rs = 0.438, *p* = 0.016^**^
LTI HA z-score	rs = −0.486, *p* = 0.006^**^	rs = 0.010, *p* = 0.958	rs = 0.356, *p* = 0.053
FTI	rs = 0.445, *p* = 0.012^**^	rs = −0.217, *p* = 0.249	rs = 0.062, *p* = 0.743
FTI z-score	rs = 0.373, *p* = 0.039^**^	rs = −0.193, *p* = 0.306	rs = 0.055, *p* = 0.772
LTI/FTI	rs = −0.450, *p* = 0.011^**^	rs = 0.265, *p* = 0.156	rs = 0.058, *p* = 0.759
Serum Ca^*^	rs = −0.184, *p* = 0.321	rs = −0.257, *p* = 0.170	rs = 0.167, *p* = 0.377
Serum P^*^	rs = 0.465, *p* = 0.008^**^	rs = 0.492, *p* = 0.006^**^	rs = 0.182, *p* = 0.335
Serum 25(OH)D^*^	rs = −0.248, *p* = 0.179	rs = −0.297, *p* = 0.135	rs = −0.014, *p* = 0.940
Serum albumin^*^	rs = −0.108, *p* = 0.565	rs = −0.097, *p* = 0.611	rs = 0.360, *p* = 0.051
Serum leptin	rs = 0.369, *p* = 0.041^**^	rs = −0.228, *p* = 0.225	rs = 0.099, *p* = 0.604

*BMI, body mass index; LTI, lean tissue index; HA, height-age; FTI, fat tissue index; Ca, calcium; P, phosphorus; 25(OH)D, 25-hydroxyvitamin D*.

* *In patients with CKD stage 4–5D the 6-month mean serum Ca, P, 25(OH)D and albumin were recorded*.

** *p statistically significant*.

**Table 3:** Mann-Whitney and univariate logistic regression analysis to identify association of mineral metabolism laboratory parameters with high fat tissue index (FTI) z-score (12 patients) in chronic kidney disease (CKD) stage 3 patients.

	**High FTI z-score 12 patients**	**Normal FTI z-score 19 patients**	***p***	**OR (95% CI)**	***p***
iPTH	97.2 (44–176)	38 (11–173)	*p* = 0.001^*^	1.024 (1.004–1.044)	*p* = 0.018^*^
Serum Ca	9.7 (8.8–10.4)	9.7 (8.8–10.3)	*p* = 0.952	1.085 (0.209–5.646)	*p* = 0.922
Serum P	4.7 (3.5–5.5)	4.2 (3.4–5.4)	*p* = 0.152	2.600 (0.669–10.107)	*p* = 0.168
Serum 25(OH)D	23.7 (18.2–28.6)	26 (12.5–37.6)	*p* = 0.141	0.903 (0.762–1.069)	*p* = 0.235

*iPTH, intact parathormone; Ca, calcium; P, phosphorus; 25(OH)D, 25-hydroxyvitamin D*.

* *p statistically significant*.

In patients with advanced CKD, 6-month mean serum iPTH was, as expected, significantly higher than serum iPTH in moderate CKD (*p* < 0.001), but no correlation was observed between serum iPTH and anthropometric parameters and body composition indices (Table 2). Moreover, serum iPTH was inversely but not significantly correlated to serum leptin (rs = −0.228, *p* = 0.225). Of note, serum leptin was positively correlated to FTI z-score (rs = 0.401, *p* = 0.028), and did not significantly differ from that in moderate CKD (*p* = 0.292).

**Figure 3:**
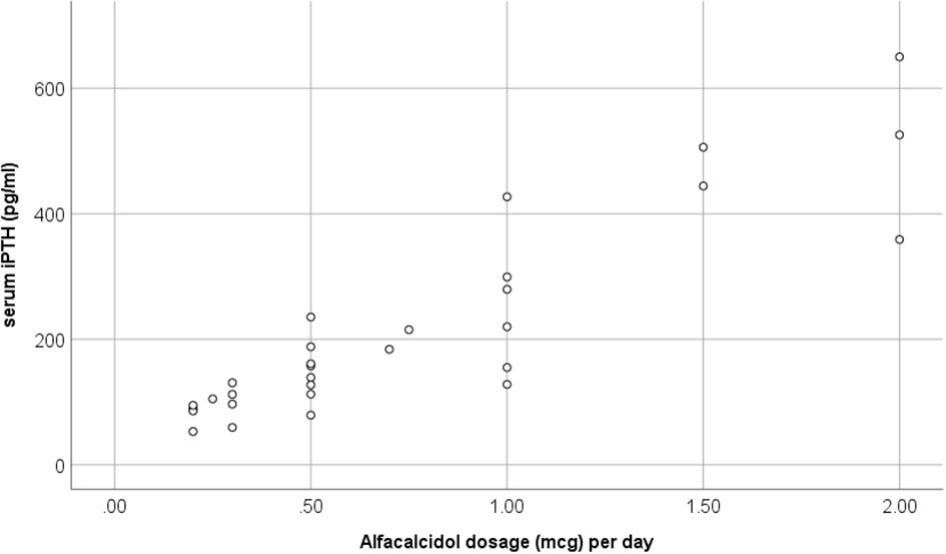
Scatter plot analysis of 6-month mean alfacalcidol dosage (mcg) per day and mean serum intact parathormone (iPTH) in chronic kidney disease (CKD) stage 4–5D patients.

**Figure 4:**
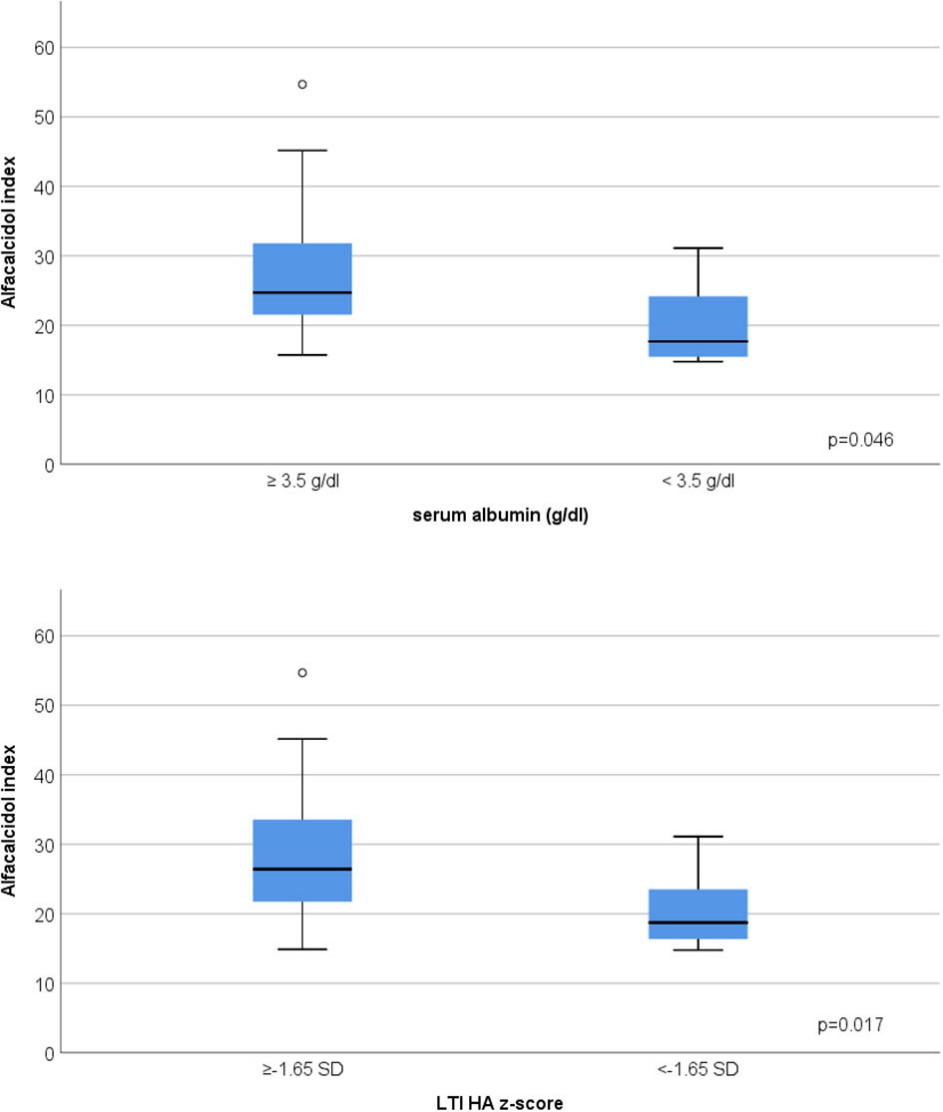
Association between low 6-month mean serum albumin (<3.5 g/dl), low lean tissue index adjusted to height-age z-score (LTI-HA) (LTI HA z-score < −1.65) and alfacalcidol index [(mcg/week per pg/ml of iPTH) × 1,000] in chronic kidney disease (CKD) stage 4–5D patients.

**Figure 5:**
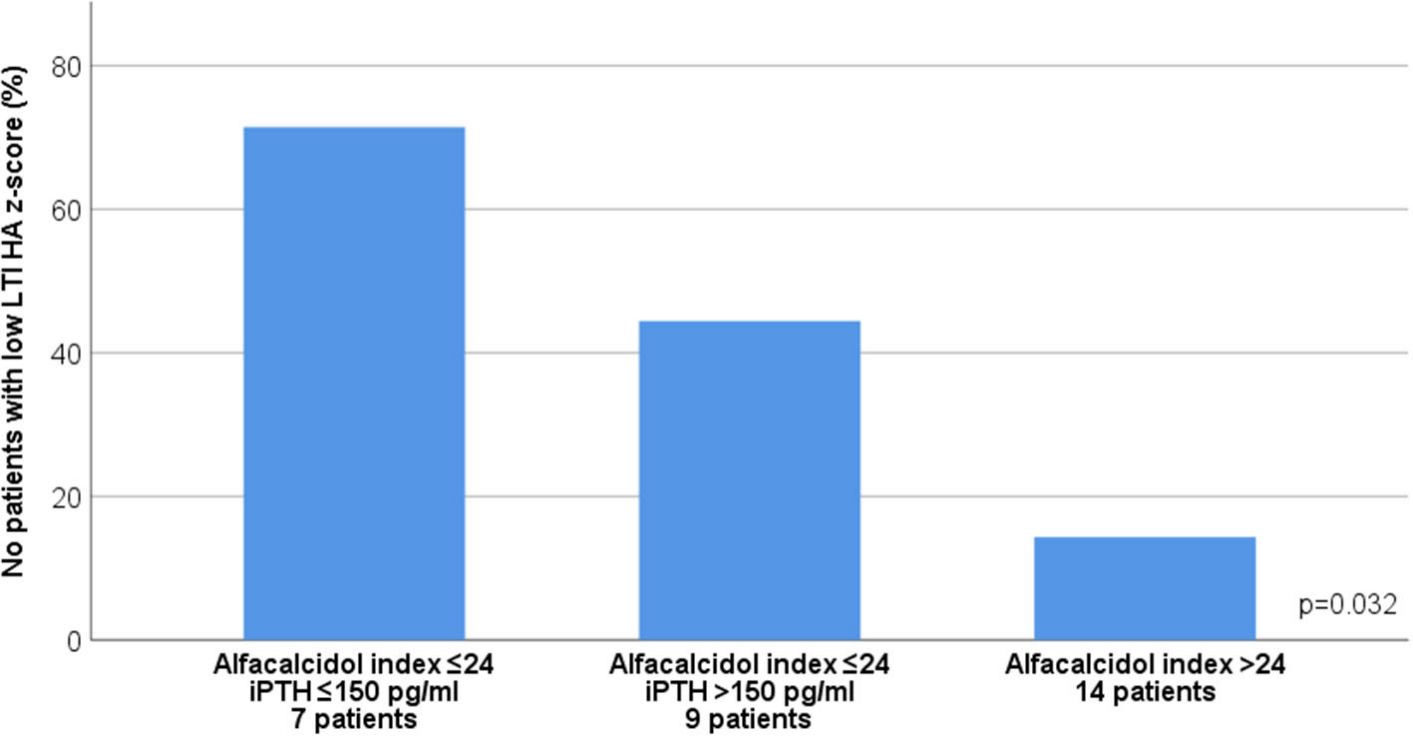
Association between 6-month mean alfacalcidol index ([mcg/week per pg/ml of iPTH] × 1000) and low lean tissue index adjusted to height-age z-score (LTI-HA) (LTI HA z-score < −1.65) after stratification by 6-month mean serum intact parathormone (iPTH) levels in chronic kidney disease (CKD) stages 4-5D patients.

In advanced CKD, alfacalcidol therapy, with a median 6-month mean daily dose of 0.5 mcg (range 0.2–2 mcg), was adapted to patient serum iPTH levels (rs = 0.876, *p* < 0.001) (Figure 3). Alfacalcidol index (median value 23.5, range 14.8–54.7) was positively correlated to weight, height and LTI z-score (rs = 0.550, *p* = 0.002, rs = 0.642, *p* < 0.001 and rs = 0.438, *p* = 0.016, respectively) (Table 2) and was significantly lower in patients with low LTI HA z-score (<-1.65 SD) (*p* = 0.017) and 6-month mean serum albumin <3.5 g/dl (8 patients, 26.7%) (*p* = 0.046) (Figure 4). In further analysis, patients were divided according to approximate median alfacalcidol index in those with alfacalcidol index >24 (14 patients) and alfacalcidol index ≤ 24 (16 patients). The patient group with low alfacalcidol index was also stratified based on 6-month mean serum iPTH level (cut off 150 ng/ml, median value). We observed that low LTI HA z-score was more prevalent in patients with alfacalcidol index ≤ 24 and mean serum iPTH ≤ 150 ng/ml (5 patients, 71.4%) or mean serum iPTH > 150 ng/ml (4 patients, 44.4%), compared to those with alfacalcidol index > 24 (2 patients, 14.3%) (*p* = 0.032) (Figure 5). Alfacalcidol index ≤ 24 was strongly associated with low LTI HA z-score (OR 7.714, 95% CI 1.284–46.364, *p* = 0.026) and the association remained significant in backward logistic regression analysis after adjustment for CKD stage and serum mineral metabolism parameters, including serum Ca, P and 25(OH)D (OR 7.226, 95% CI 1.150–45.384, *p* = 0.035).

## Discussion

Pediatric CKD is commonly complicated by mineral and bone disorder (CKD-MBD), which mainly involves abnormal mineralization and disturbed bone turnover (27, 28). SHPT is the principal laboratory parameter of CKD-MBD, which is highly prevalent in advanced stages, but it may also occur earlier in the course of the disease. In our study, 32.3% of patients with moderate and 100% of patients with advanced CKD presented high serum iPTH levels. Additional to glomerular filtration rate deterioration, disturbed calcium, phosphorus and vitamin D balance constitute the principal risk factor of SHPT. As expected, in the current study, serum iPTH levels were significantly correlated to serum P in patients with either moderate or advanced CKD. Apart from controlling bone health, mineral homeostasis is involved in the regulation of various physiological processes, including renin angiotensin system (29), cardiovascular function (30), and bone hematopoiesis (31), while the interaction between mineral imbalance and other body system functions in the setting of CKD has recently gained research interest. Our study focuses on exploring the crosstalk between SHPT and body muscle and fat status in children with moderate and advanced CKD.

Body composition assessment is crucial for nutritional evaluation of pediatric patients with CKD. In our study, we applied BIS technique for estimation of body composition, and we used LTI-HA and FTI as indices of body muscle and fat status. We observed that prevalence of muscle wasting increased, while prevalence of high adiposity reduced from moderate to advanced CKD. Moreover, low adiposity, the prevalence of which is in general undetermined in pediatric CKD, was present in 10% of patients with advanced CKD.

In the current study, we found that, in moderate CKD, patients with SHPT presented higher FTI z-score levels, serum iPTH was positively correlated to FTI z-score and was independently associated with high adiposity. Although, obesity was associated with a higher risk of SHPT in pre-dialysis CKD adult patients (16, 17), to our knowledge, this association has not been described yet in pediatric population. Multiple pathogenetic mechanisms may explain this association. It is widely known that serum 25(OH)D levels are reduced in case of obesity in both adult and pediatric population (32, 33), primarily because of vitamin D sequestration or volumetric dilution in large body fat compartments, leading to decreased bioavailability from cutaneous and dietary sources (34, 35). In addition, sedentary lifestyle of subjects with high body adiposity levels, involving reduced sun ultraviolet B exposure, limited outdoor activities and inadequate mineral intake from unhealthy high caloric food, may aggravate hypovitaminosis D in this population (36). In pre-dialysis CKD, hypovitaminosis D is more prevalent in overweight patients in both adult and pediatric patients (37, 38). In our study, as expected, patients with SHPT presented lower serum 25(OH)D levels (*p* = 0.159), serum iPTH was negatively correlated to serum 25(OH)D levels (rs = −0.248, *p* = 0.179), and serum 25(OH)D levels were lower in patients with high FTI z-score level (*p* = 0.141), but none of these correlations reached statistical significance. Besides, the association between serum iPTH and high adiposity remained significant after adjustment for other serum mineral metabolism parameters, suggesting a direct crosstalk between adipose tissue and parathyroid gland. Experimental *in vivo* and *in vitro* studies have evocated that adipose tissue derived leptin stimulates PTH excretion either indirectly, through upregulation of bone fibroblast growth-factor 23 expression in parallel with downregulation of kidney calcitriol production, or even by direct stimulatory action of PTH secretion from parathyroid gland (39, 40). In clinical studies, serum leptin levels were positively correlated to serum PTH levels in both general obese adult and pediatric population (41, 42). In our study, we remarked a broad range of serum leptin levels which accords with the large variety of FTI values observed in our population. As expected, serum leptin levels were positively FTI z-score. Moreover, serum leptin levels were higher in patients with SHPT and were positively correlated to serum iPTH, while the correlation between serum iPTH and FTI z-score lost significance after adjustment for serum leptin. These findings may suggest that high adiposity could enhance the risk of SHPT occurrence in moderate CKD and serum leptin may contribute to their association. Further large-scale studies are needed to confirm these results.

The association between serum leptin and SHPT has been explored in adult CKD population but the results are inconsistent among different studies (43), where an inverse (44), positive (45) or absence of correlation (46) between the two laboratory parameters was evocated. Moreover, according to an *in-vitro* study on differentiated adipocytes from humans with severe SHPT, high PTH levels suppressed adipocyte leptin production *via* inhibition of Akt signaling, indicating a negative effect of PTH on adipocyte leptin secretion (25). In our study, although serum iPTH was positively correlated to serum leptin in moderate CKD, an inverse non-statistically significant correlation between the two parameters was observed in advanced CKD patients. Of note, while serum leptin was positively correlated to FTI z-score, the strength of correlation was attenuated in patients with advanced CKD, suggesting that apart from fat mass, other factors may contribute to serum leptin expression in late CKD stages, such as inflammatory cytokine expression (47). Further studies are needed to explore the impact of PTH on serum leptin and the potential contributive role of inflammatory cytokine profile on their correlation in advanced CKD.

Few clinical adult studies have investigated the relation between serum PTH and body composition in advanced CKD. Most clinical studies suggest that serum PTH may serve as a nutritional marker, since it is positively correlated to both fat and muscle mass (48) especially in male population, it generally decreases along with decline of BMI (21), while patients with serum PTH <150 pg/ml present more frequently markers of protein-energy wasting and inflammation, involving reduced muscle mass and serum albumin levels, and generally receive lower active vitamin D analogs (20). In our study, no significant correlation was observed between 6-month mean serum iPTH and anthropometric parameters or body composition indices in patients with advanced CKD. Nevertheless, we found that the ratio of alfacalcidol weekly dose per each unit of serum iPTH, defined as alfacalcidol index, was lower in patients with muscle wasting and low serum albumin, while alfacalcidol index ≤ 24 was significantly associated with muscle wasting after adjustment for other serum mineral metabolism parameters. Of note, no correlation was observed between alfacalcidol index and FTI. In adult patients on chronic hemodialysis, Shinaberger et al., mentioned that a higher weekly paricalcitol dosage per each unit of serum PTH was associated with a progressive greater survival (49). The benefits of a higher ratio of vitamin D receptor activator dosage to serum PTH in terms of patient nutritional status has never been previously explored. In our study, patients with lower alfacalcidol index consisted in those with lower serum iPTH levels and subsequently lower alfacalcidol dose and in those with higher serum iPTH levels inadequately controlled by alfacalcidol treatment. Both patient groups presented higher muscle wasting prevalence (71.4 and 44.4%, respectively) compared to those with higher alfacalcidol index (14.3%). It is probable that in the first patient group, malnutrition is the main cause of lower alfacalcidol index, and therefore muscle wasting, in accordance with the findings from clinical adult studies previously described. In the second patient group, we speculate that the relatively high serum iPTH levels coupled with relatively low alfacalcidol dosage are responsible for the remarked reduced LTI. As already mentioned, SHPT has been recently incriminated for muscle wasting, according to the results of *in-vivo* studies (22, 23). Furthermore, vitamin D deficiency is generally considered as a contributor factor of muscle wasting in both general population and CKD (50) while native and active vitamin D supplementation seem to reduce adipose tissue browning and muscle wasting, by modulating the expression of thermogenic genes on a mouse model of CKD-associated cachexia (51). Therefore, we presume that both lower serum iPTH levels due to malnutrition and higher serum iPTH levels coupled with insufficient active vitamin D analog dosage are associated with poor muscle status in pediatric advanced CKD.

Optimal PTH control in advanced CKD remains a cornerstone challenge for pediatric nephrology in clinical practice. The benefits of active vitamin D analogs for reducing serum PTH levels are counterweighted by the risk of rising serum calcium levels and subsequently calcium-phosphate product, eventually leading to extraskeletal calcifications and worsening of cardiovascular disease. Calcimimetics and new oral phosphate binders, such as sucroferric oxyhydroxide, which are currently approved for administration in pediatric population, have been proven effective for serum phosphorus and PTH management (52, 53). The benefits of these drugs on the preservation of muscle mass in advanced pediatric CKD needs to be answered in the future.

Our study has some limitations. Firstly, the small number of included patients of various ages with a broad spectrum of CKD severity preclude us from making definite conclusions. Moreover, the limited number of recruited patients with CKD 4 and CKD 5 and 5D did not allow us to perform a further subgroup data analysis for each CKD stage patient groups. In addition, the impact of pubertal status on PTH values and therefore on the association between PTH and body composition indices was not taken into account in our study. Furthermore, adjustment of serum iPTH to alfacalcidol dosage without considering other medications, which may have also influenced serum iPTH levels, such as oral phosphate binders, calcimimetics, native vitamin D and calcium supplement, limits the strength of our results. Finally, the cross-sectional nature of the study does not allow us to safely determine the cause-effect relationships of the on-study associations.

## Conclusion

In conclusion, our study showed that pediatric patients with high adiposity may present secondary hyperparathyroidism earlier in the course of CKD, suggesting a direct link between adipose tissue and parathyroid gland. The potential benefits of lowering high adiposity in preventing secondary hyperparathyroidism in children and adolescents with moderate CKD needs further investigation. Moreover, we observed that pediatric patients with advanced CKD who maintain low serum iPTH levels with low alfacalcidol dosage and those who preserve high serum iPTH despite active vitamin D analog therapy present higher frequency of muscle wasting, suggesting that alfacalcidol index may serve as a marker of evaluation of nutritional status in everyday clinical practice. Additional large-scale prospective studies are required to explore the prognostic value of alfacalcidol index on estimating the progression of muscle wasting in children under active vitamin D analogs.

## Data Availability Statement

The original contributions presented in the study are included in the article/supplementary material, further inquiries can be directed to the corresponding author/s.

## Ethics Statement

The studies involving human participants were reviewed and approved by Committee for Bioethics and Ethics of School of Medicine of Aristotle University of Thessaloniki. Written informed consent to participate in this study was provided by the participants' legal guardian/next of kin.

## Author Contributions

NP, AC, ES, and VK conceived and designed the analysis of the study. VK, AK, JD, and KK collected the data. AT and KT performed the laboratory tests. VL provided the BIS device. VK, AK, and JD performed the statistical analysis. VK wrote the paper. AC, EF, ES, and VL reviewed the paper. NP made the final revision. All authors contributed to the article and approved the submitted version.

## Conflict of Interest

The authors declare that the research was conducted in the absence of any commercial or financial relationships that could be construed as a potential conflict of interest.

## Publisher's Note

All claims expressed in this article are solely those of the authors and do not necessarily represent those of their affiliated organizations, or those of the publisher, the editors and the reviewers. Any product that may be evaluated in this article, or claim that may be made by its manufacturer, is not guaranteed or endorsed by the publisher.
